# The efficacy of cognitive behavioral therapy on reducing suicidal symptoms among adults: a systematic review and meta-analysis

**DOI:** 10.3389/fpsyg.2025.1672957

**Published:** 2025-10-14

**Authors:** Feng Tong, Yunxu Zhang, Yang Jiao

**Affiliations:** ^1^International School of Law and Society, Sichuan International Studies University, Chongqing, China; ^2^College of Arts, Sichuan University, Chengdu, China

**Keywords:** cognitive behavioral therapy, suicidal ideation, self-harming behavior, systematic review, meta-analysis

## Abstract

**Objective:**

This study aimed to systematically evaluate the effectiveness of cognitive behavioral therapy (CBT) on alleviating suicidal ideation, suicidal and self-harming behaviors, and depressive symptoms in adults.

**Methods:**

Comprehensive searches were conducted in both English and Chinese databases including PubMed, PsycINFO, Cochrane Library, Web of Science, Embase, Scopus, CINAHL, HSE, ProQuest, CNKI, and Wanfang. Randomized controlled trials (RCTs) involving adults aged 18–65 years receiving CBT for suicidal symptoms were included. The primary outcome was suicidal ideation, while secondary outcomes included suicidal and self-harming behaviors and depressive symptoms. Risk of bias was assessed using the Cochrane Risk of Bias tool, and meta-analyses were conducted using a random-effects model. Subgroup analyses were performed based on follow-up duration (short-term ≤6 months, mid-term 6–12 months, long-term >12 months). Outcomes were reported using standardized mean differences (SMDs), odds ratios (ORs), and 95% confidence intervals (CIs).

**Results:**

A total of 28 RCTs (*n* = 5,883) were included. In the short term, CBT significantly reduced suicidal ideation (SMD = −0.25, 95% CI: −0.34 to −0.16); however, no significant effects were observed at mid-term (SMD = −0.06, 95% CI: −0.24 to 0.12) or long-term (SMD = −0.18, 95% CI: −0.41 to 0.05) follow-up. CBT significantly reduced the risk of suicidal and self-harming behaviors across all follow-up durations (short-term OR = 0.72, 95% CI: 0.53 to 0.97; mid-term OR = 0.73, 95% CI: 0.54 to 0.98; long-term OR = 0.50, 95% CI: 0.31 to 0.80). CBT was also more effective than controls in improving depressive symptoms across all time points (short-term SMD = −0.36, 95% CI: −0.50 to −0.22; mid-term SMD = −0.26, 95% CI: −0.46 to −0.05; long-term SMD = −0.39, 95% CI: −0.56 to −0.21), with statistically significant differences.

**Conclusion:**

Cognitive behavioral therapy shows significant short-term benefits in reducing suicidal ideation and sustained effects in reducing suicidal/self-harming behaviors and improving depressive symptoms among adults. CBT may serve as an effective psychological intervention for suicide prevention in adults, although its long-term impact warrants further investigation.

## 1 Introduction

Suicide has emerged as a critical global public health concern. According to the World Health Organization, approximately 727,000 individuals die by suicide each year worldwide (World Health Organization, 2025). Among young people aged 15–29, suicide is the third leading cause of death, and it poses a significant disease burden among adults aged 18–65 (Xie et al., 2025). Suicidal ideation and behaviors exert a profound impact not only on individuals and families but also on society at large, creating substantial social and economic costs (World Health Organization, 2025). Cognitive Behavioral Therapy (CBT), a structured and problem-oriented form of psychotherapy, has attracted considerable attention in suicide prevention research for its ability to target maladaptive cognitive and behavioral patterns (Brown et al., 2005; National Collaborating Centre for Mental Health, 2012; Wenzel et al., 2009). By identifying and reconstructing suicide-related negative cognitions and enhancing emotional regulation and problem-solving skills, CBT has demonstrated potential in reducing suicide risk. Over time, CBT has evolved into various modalities, including first-wave Behavioral Therapy (BT) (Clark and Fairburn, 1997), second-wave Cognitive Therapy (CT), classic CBT and its brief versions (e.g., Manual-Assisted Cognitive Therapy [MACT], Brief CBT [BCBT]), internet-based CBT (iCBT) (Andersson et al., 2014; Clark and Beck, 1999; Davidson et al., 2014), as well as third-wave approaches such as Mindfulness-Based Cognitive Therapy (MBCT), Dialectical Behavior Therapy (DBT), Acceptance and Commitment Therapy (ACT) (Hayes et al., 2006; Morgan, 2003; Robins and Rosenthal, 2011), and Compassion-Focused Therapy (CFT) (Gilbert, 2010). Meanwhile, CBT has also been integrated with other disciplinary frameworks, leading to the emergence of hybrid approaches such as Cognitive-Behavioral Art Therapy (CB-AT) (Czamanski-Cohen et al., 2014; Johnson and Johnson, 2009).

Despite CBT’s widely recognized potential in mitigating suicide risk, its efficacy remains a subject of debate. Some primary studies reported no significant reduction in suicidal ideation following CBT (Baker et al., 2024; Tarrier et al., 2014; Weitz et al., 2014), whereas others (e.g., Nazem, Yang) found CBT effective in reducing suicidal ideation, depression, and social distress within short-term follow-ups (≤6 months) (Nazem et al., 2024; Yang et al., 2025), though these benefits declined over time and suicide attempt rates did not show statistical significance. Cottraux et al. (2009) and Rudd et al. (2015) reported that the positive effects of CBT may persist for over 1 year. Among published systematic reviews, Mewton and Andrews (2016) found only partial evidence supporting CBT’s efficacy in reducing suicidal thoughts and behaviors. Ghazal et al. (2025) focusing on adolescents, concluded that about half of the trials showed no significant difference between CBT and control groups. Büscher et al. (2022) found that CBT significantly reduced suicidal ideation in the short term, but there was insufficient data to evaluate its effects on suicidal or self-harming behaviors. Pedrola-Pons et al. (2024) demonstrated that CBT was effective in reducing suicidal ideation among incarcerated individuals at high risk of suicide.

Various forms and Characteristic Modules of cognitive behavioral therapy (CBT) are currently applied in suicide prevention, each offering distinct mechanisms and clinical advantages, among them cognitive restructuring and behavioral experiments were core modules. Classical CBT is the earliest and most widely validated form, utilizing structured techniques such as thought records, behavioral experiments, and problem-solving training to systematically address suicide-related cognition and impulsivity (Davidson et al., 2006; Husain et al., 2023; Linehan et al., 1991). Simplified or specialized adaptations of CBT, including Brief CBT (BCBT), Manual-Assisted CBT (MACT), and CBT for Suicide Prevention (CBT-SP), prioritize rapid risk reduction. These models often incorporate elements such as suicide event chain analysis, safety planning, and focused skills training. Designed for immediate crisis response, they are particularly suited for use in emergency or acute psychiatric care settings (Linehan et al., 1991; De Jaegere et al., 2019). Internet-based CBT (iCBT) delivers therapeutic content via online platforms and offers several practical advantages: cost-effectiveness, high accessibility, and user anonymity. Evidence suggests that iCBT can significantly reduce suicidal ideation across a wide range of demographic groups—regardless of age, gender, or history of suicide attempts. This makes it especially valuable in settings with limited mental health resources, for patients who face barriers to accessing in-person care, or when urgent interventions are required (Nazem et al., 2024; Büscher et al., 2022). Third-wave CBT approaches, such as Dialectical Behavior Therapy (DBT), Acceptance and Commitment Therapy (ACT), and Mindfulness-Based Cognitive Therapy (MBCT), each have unique theoretical foundations and target populations. DBT emphasizes emotion regulation and interpersonal effectiveness, and is particularly effective for self-harm in patients with borderline personality disorder (Torok et al., 2022). ACT aims to reduce the impact of pain on behavior through “acceptance” and cognitive defusion. Some studies have shown that ACT can lower suicidal ideation, but due to limited quantity and quality of research, current evidence is insufficient for it to be recommended as a stand-alone suicide intervention (Jobes, 2023; Tighe et al., 2018; Twohig and Levin, 2017). MBCT integrates mindfulness training with cognitive therapeutic principles to enhance emotional awareness, reduce negative automatic thoughts and rumination, and support relapse prevention. It has demonstrated particular value in reducing suicidal ideation among individuals with recurrent depression or at high risk of suicide (Cladder-Micus et al., 2018; Qaseem et al., 2023; Zhang et al., 2022).

In summary, each form of CBT offers specific strengths in suicide prevention. Tailoring and integrating these approaches according to the clinical context and the individual needs of patients may help optimize therapeutic outcomes and expand access to effective interventions. The inconsistency of these findings suggests that while some studies support short-term efficacy, others underscore the lack of robust data. Furthermore, most existing systematic reviews have primarily focused on adolescents, with few targeting adult populations exclusively. Additionally, most reviews did not conduct stratified analyses based on follow-up duration (short-term, mid-term, long-term), leaving the sustainability of CBT’s effects underexplored.

In light of these gaps, this study aims to systematically review and meta-analyze the efficacy of CBT in reducing suicidal ideation and behaviors in adults (aged 18–65), with particular attention to stratified effects across different follow-up durations. The goal is to provide timely and targeted evidence to inform clinical practice, policy development, and future intervention research, thereby contributing to the optimization of suicide prevention strategies.

## 2 Methods

### 2.1 Literature search

A comprehensive search was conducted across the following databases: PubMed, PsycINFO, Cochrane Library, Web of Science, Embase, Scopus, CINAHL, Health Systems Evidence (HSE), ProQuest, China National Knowledge Infrastructure (CNKI), and Wanfang Data. The search covered all available publications up to May 6, 2025. Additionally, the reference lists of included studies were manually screened for relevant articles. Both subject terms and free-text keywords were employed. The English search strategy included the following terms: (suicid*, suicidal ideation, suicidal behavior, suicidal behavior, self-harm, self-injury) AND (cognitive behavioral therapy, cognitive behavioral therapy, cognitive therapy, behavioral therapy) AND (randomized controlled trials). For Chinese databases, search terms included: (“自杀”) AND (“认知行为治疗,” “认知行为疗法,” “认知治疗,” “认知疗法,” “行为治疗,” “行为疗法”) AND (“随机对照研究”).

### 2.2 Study selection and quality assessment

This systematic review followed the PRISMA 2020 guidelines (Preferred Reporting Items for Systematic Reviews and Meta-Analyses) for study identification, selection, and reporting (Page et al., 2021). Two independent reviewers screened studies based on predefined inclusion and exclusion criteria. Discrepancies were resolved through discussion or consultation with a third reviewer. The study selection process was documented using a PRISMA flow diagram. The quality of included studies was assessed using the Cochrane Risk of Bias tool (RoB 2), which evaluates several domains, including sequence generation, allocation concealment, blinding, completeness of outcome data, and selective reporting (Sterne et al., 2019).

If a study contained multiple eligible intervention arms, the group with the most comprehensive CBT content and longest duration was selected. For studies with multiple control groups, the treatment-as-usual (TAU) group was prioritized; otherwise, the most effective comparator was used.

The primary outcome was suicidal ideation, while secondary outcomes included suicidal or self-harming behaviors and depressive symptoms. Suicidal ideation was preferably measured using the Beck Scale for Suicide Ideation (BSS); if unavailable, other validated tools such as the Adult Suicidal Ideation Questionnaire (ASIQ), Beck Hopelessness Scale (BHS), or proxy indicators were used. Depression was primarily assessed using the Beck Depression Inventory (BDI) or the Hamilton Rating Scale for Depression (HRSD). Any other scales used in the included studies were also recorded to ensure transparency and comparability. The review adhered strictly to PRISMA 2020 protocols to ensure reproducibility and methodological rigor.

### 2.3 Inclusion criteria

Studies were included if they met the following criteria: (1) Published in English or Chinese as a randomized controlled trial (RCT); (2) Included adults aged 18–65 years; (3) Participants exhibited suicidal ideation or suicidal behavior at baseline; (4) The intervention group received CBT or CBT-integrated therapies with a clear structure, incorporating cognitive, behavioral, or combined approaches (including brief CBT versions such as MACT, BCBT), and core techniques such as cognitive restructuring, behavioral activation, problem-solving, social skills training, and relaxation techniques. Third-wave CBT modalities such as ACT, DBT, and MBCT were also included; (5) The control condition was unrestricted (e.g., TAU, placebo, waitlist); (6) At least one outcome related to suicidal ideation, suicidal behavior, or self-harm was reported.

### 2.4 Exclusion criteria

Studies were excluded based on the following: (1) Non-RCT designs or quasi-randomized designs (e.g., allocation by hospital number); (2) Conference abstracts or dissertations lacking complete data or methods; (3) Studies labeled as CBT but lacking core cognitive or behavioral techniques; (4) Sample size in any group < 20 participants; (5) Participants with end-stage physical illnesses where suicide was primarily driven by physical disease; (6) No quantitative data reported for suicidal ideation, behavior, or self-harm; (7) Full text unavailable or missing critical data; (8) Duplicate reports—only the most complete version was included.

### 2.5 Statistical analysis

Meta-analyses were performed using a random-effects model. For dichotomous outcomes, odds ratios (ORs) with 95% confidence intervals (CIs) were calculated. For continuous outcomes, mean differences (MDs) or standardized mean differences (SMDs) were used depending on scale consistency. Primary outcomes were analyzed according to the intention-to-treat (ITT) principle when reported. Missing or incomplete data were handled as described in the original studies. Subgroup analyses were conducted based on follow-up duration: short-term (≤6 months), mid-term (6–12 months), and long-term (>12 months). To preserve the independence required for two-level meta-analysis, when a study reported multiple assessments within the same window, a single representative assessment was selected *a priori* according to the following rules: priority was given to a suicidality-specific instrument aligned with the primary outcome; if multiple time points were available within the window, the assessment closest to the upper bound of that window was retained. Data from the same assessment time point were not permitted to be assigned to adjacent windows. Heterogeneity was assessed using Cochran’s *Q* test and the *I*^2^ statistic, with *I*^2^ > 50% or *p* < 0.10 indicating significant heterogeneity. Where applicable, sensitivity analyses were conducted to explore heterogeneity sources. All analyses were performed using RevMan 5.3 software, with *p* < 0.05 considered statistically significant.

## 3 Results

### 3.1 Literature selection

**Figure 1:**
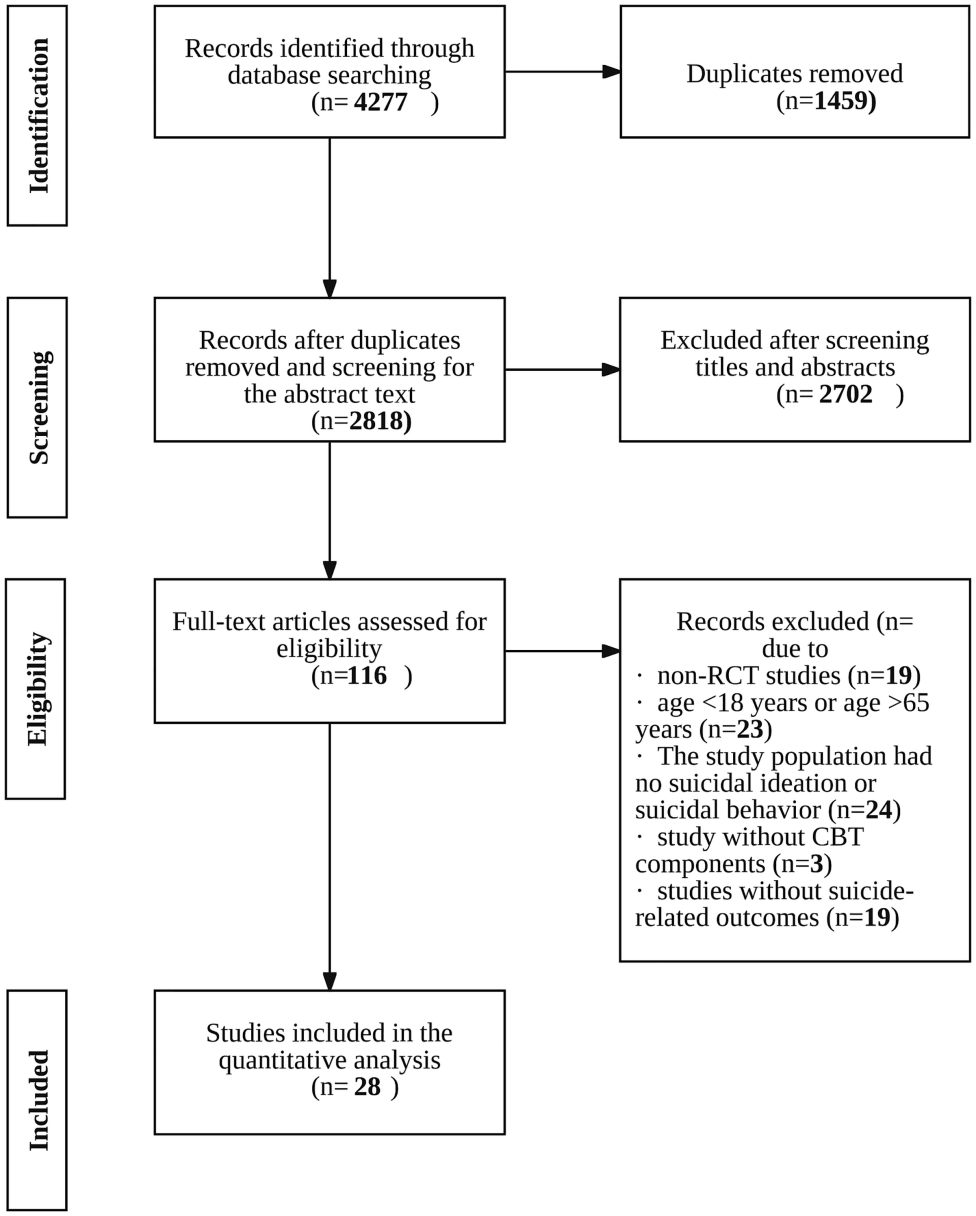
Flowchart of study identification, screening, eligibility, and inclusion.

A total of 4,277 articles were retrieved. After removing duplicates, 2,818 records remained. Following title and abstract screening, 116 full-text articles were assessed for eligibility, and ultimately, 28 randomized controlled trials (RCTs) were included in the final analysis. The study selection process is illustrated in Figure 1. In total, the 28 RCTs involved 5,883 participants from 11 countries, including the United Kingdom, United States, the Netherlands, France, Australia, Germany, China, Belgium, Denmark, Pakistan, and South Korea. Sample sizes ranged from 30 participants to 901. Intervention types included classic CBT, MACT, MBCT, CT, iCBT, and BCBT, with the number of modules ranging from 3 to 24. Follow-up durations ranged from 6 weeks to 24 months, with most studies reporting 3–6 months follow-up periods. Detailed study characteristics are presented in Table 1.

**Table 1:** Study characteristics.

Source	Country	Population	Age at baseline, mean (SD)	Total No. at baseline (Female %)	Intervention type (no of modules /duration)	Control condition	Measure of suicide	Dropout rate, %	
								Intervention group*	Control group*
Baker et al. (2024)	USA	Adults with suicidal ideation	31.8 (12.6)	96(66.7%)	BCBT (3/12 weeks)	PCT	BSS、Suicide or self-harm episodes (n)	42.30	41.30
Brown et al. (2005)	USA	Adults who recently attempted suicide	35.0 (10.3)	120 (60.8%)	CT (10/—)	TAU	BSS	16.67	13.33
Cottraux et al. (2009)	France	Adult outpatients with borderline personality disorder and suicidal ideation	33.5 (9.3)	65 (76.9%)	CT (10/24 weeks)	RST	BHS, SHBCL	69.70	65.63
Davidson et al. (2006)	UK	Adult outpatients with borderline personality disorder and suicidal ideation	—	102 (74.5%)	CBT + TAU (Non-modular/48 weeks)	TAU	Suicide or self-harm episodes (n)	12.96	17.31
De Jaegere et al. (2019)	Belgium	Adults with suicidal ideation	35.7(13.6)	724(59.4%)	CBT, DBT, PST, MBCT (6/6 weeks)	Wait-list	BSS	74.00	52.10
De Jaegere et al. (2024)	Belgium	Adults with suicidal ideation	43.9(12)	93(72%)	MBCT (8/8 weeks)	TAU	BSS	55.41	31.58
Diefenbach et al. (2024)	USA	Adults who recently attempted suicide	32.8(12.6)	200(58.5%)	BCBT (4/2 weeks)	TAU	ASIQ	43.60	42.50
Fischer et al. (2015)	Germany	Adult patients with multiple sclerosis and suicidal ideation	45.3 (11.6)	90 (77.8%)	iCBT (10/9 weeks)	Wait-list	SBQ-R、BDI item 9	22.22	20.00
Forkmann et al. (2016)	Germany	Adult with suicidal ideation	50.8 (11.9)	106 (62.3%)	MBCT (9/8 weeks)	TAU	HAMD item 3、BDI item 9	—	
Fu and Li (2016)	China	Patients with depressive disorder and suicidal ideation	43.2 (9.5)	66 (63.6%)	CBT + Normal medication (6/12 weeks)	Normal medication	BSI-CV	0.00	0.00
Gandy et al. (2014)	Australia	Adult patients with epilepsy and suicidal ideation	39.3 (12.6)	59(—)	CBT (9/9 weeks)	Wait-list	NDDI-E	38.71	17.86
Husain et al. (2023)	Pakistan	Adults with suicidal ideation	26.5(7.97)	901(60.4%)	CBT (6/12 weeks)	TAU	BSS	3.60	6.50
Morley et al. (2014)	Australia	Adults with suicidal thoughts	36.0 (11.2)	185 (36.8%)	iCBT (12/24 weeks)	TAU	BSS	63.93	52.38
Mühlmann et al. (2021)	Denmark	Adults with suicidal ideation	33.6(13)	402(70.9%)	iCBT (6/6 weeks)	Wait-list	BSS、SIDAS	8.67	19.42
Nazem et al. (2024)	USA	Veterans with Insomnia and suicidal ideation	38.8(7.6)	50(30%)	iCBT (6/9 weeks)	Insomnia education	ASIQ	—	
Palmier-Claus et al. (2025)	UK	University students with suicidal ideation	21.3(3.4)	64(75%)	CT (6/—)	TAU	BSS、Suicide or self-harm episodes (n)	15.20	3.20
Rudd et al. (2015)	USA	Active-duty Army soldiers with suicidal thoughts	27.4 (6.2)	152 (12.5%)	BCBT (3/—)	TAU	BSS	73.68	64.47
Slee et al. (2008)	Netherlands	Adults with recent episodes of severe self-harm	24.6 (5.5)	90 (94%)	CBT + TAU (12/22 weeks)	TAU	BSS、Suicide or self-harm episodes (n)	16.67	21.43
Tarrier et al. (2014)	UK	Adult patients with schizophrenia spectrum disorders and suicidal ideation	34.9 (13.1)	49 (36.7%)	CBT + TAU (24/12 weeks)	TAU	BSS、ASIQ	32.00	25.00
Torok et al. (2022)	Australia	young adults with suicidal ideation	21.5(2.18)	455(84.6%)	DBT (7/8 weeks)	TAU	SIDAS	48.20	40.10
Tyrer et al. (2003)	UK	Adult with recurrent deliberate self-harm	32.0(11)	480 (32%)	MACT +TAU (Non-modular /12 weeks)	TAU	BHS、Suicide or self-harm episodes (n)	10.88	9.96
van Spijker et al. (2014)	Netherlands	Adult with suicidal ideation	40.9 (13.7)	236 (66.1%)	iCBT, DBT, PST, MBCT (6/6 weeks)	Wait-list	BSS、Suicide or self-harm episodes (n)	9.50	8.30
van Spijker et al. (2018)	Australia	Adults with suicidal thoughts	40.6 (11.9)	418 (77.3%)	iCBT, DBT, PST, MBCT (6/6 weeks)	Attention control	C-SSRS	43.50	48.30
Walton et al. (2020)	Australia	Adults with suicidal ideation	26.6(7.8)	162(77%)	DBT (8/8 weeks)	TAU	Suicide or self-harm episodes (n)	35.00	25.00
Weinberg et al. (2006)	USA	Adult with recurrent deliberate self-harm	28.2 (8.2)	30 (100%)	MACT +TAU (6/6–8 weeks)	TAU	SBQ-R、 Suicide or self-harm episodes (n)	0.00	13.33
Weitz et al. (2014)	USA	Patients with major depressive disorder and suicidal ideation	35.0 (10.0)	239 (70%)	CBT (Non-modular/16 weeks)	Placebo+CM	HRSD item 3、BDI item 9	—	
Wilks et al. (2018)	USA	Suicidal individuals with heavy episodic drinking and emotion dysregulation	38(10.4)	59(69.5%)	DBT (8/8 weeks)	Wait-list	BSS	26.70	3.40
Yang et al. (2025)	Korea	Patients with depressive disorder and suicidal ideation	26.3(9.2)	190(60.5%)	iCBT+TAU (5/6 weeks)	TAU	C-SSRS	59.80	67.70

BCBT: Brief CBT; PCT: Present-Centered Therapy; BSS: Beck Scale for Suicide Ideation; CT: Cognitive Therapy; TAU: Treatment as Usual; RST: Rogerian Supportive Therapy; BHS: Beck Hopelessness Scale; SHBCL: Self-harming Behavior Checklist; CBT: Cognitive Behavioral Therapy; MBCT: Mindfulness-Based Cognitive Therapy; ASIQ: Adult Suicidal Ideation Questionnaire; iCBT: Internet-based CBT; SBQ-R: Suicide Behaviors Questionnaire-Revised; MACT: Manual-assisted Cognitive Therapy; C-SSRS: Columbia-Suicide Severity Rating Scale; “—”: indicates no information provided. *This refers to drop-out at postintervention as reported in the original trials.

### 3.2 Risk of bias assessment of included studies

A total of 28 randomized controlled trials were included in this study, and a systematic assessment of risk of bias was performed (see Table 2). Among these, 25 studies (Brown et al., 2005; Baker et al., 2024; Tarrier et al., 2014; Yang et al., 2025; Cottraux et al., 2009; Rudd et al., 2015; Husain et al., 2023; De Jaegere et al., 2019; Torok et al., 2022; Davidson et al., 2006; De Jaegere et al., 2024; Diefenbach et al., 2024; Fischer et al., 2015; Forkmann et al., 2016; Fu and Li, 2016; Gandy et al., 2014; Morley et al., 2014; Mühlmann et al., 2021; Palmier-Claus et al., 2025; Slee et al., 2008; Tyrer et al., 2003; van Spijker et al., 2014; van Spijker et al., 2018; Walton et al., 2020; Wilks et al., 2018) clearly described the method of random sequence generation, while the remaining studies did not provide sufficient detail. Twenty-four studies (Brown et al., 2005; Baker et al., 2024; Tarrier et al., 2014; Yang et al., 2025; Cottraux et al., 2009; Rudd et al., 2015; Husain et al., 2023; De Jaegere et al., 2019; Torok et al., 2022; Davidson et al., 2006; Diefenbach et al., 2024; Fischer et al., 2015; Forkmann et al., 2016; Fu and Li, 2016; Gandy et al., 2014; Morley et al., 2014; Mühlmann et al., 2021; Palmier-Claus et al., 2025; Slee et al., 2008; Tyrer et al., 2003; van Spijker et al., 2014; van Spijker et al., 2018; Walton et al., 2020; Wilks et al., 2018) reported allocation concealment, indicating good group control prior to blinding. Due to the nature of psychological intervention studies, double-blinding of participants and researchers was rarely feasible; only 6 studies (Baker et al., 2024; Yang et al., 2025; Husain et al., 2023; Torok et al., 2022; Palmier-Claus et al., 2025; Walton et al., 2020) were rated as low risk for blinding of participants and personnel, and 12 studies (Brown et al., 2005; Tarrier et al., 2014; Yang et al., 2025; Cottraux et al., 2009; Rudd et al., 2015; Husain et al., 2023; Torok et al., 2022; Davidson et al., 2006; De Jaegere et al., 2024; Diefenbach et al., 2024; Palmier-Claus et al., 2025; Walton et al., 2020) used blinded outcome assessment, with the rest being unclear in this domain.

**Table 2:** Risk of bias assessment of included studies.

Study	Random sequence generation	Allocation concealment	Blinding of participants and personnel	Blinding of outcome assessment	Incomplete outcome data	Selective reporting	Other bias	Overall risk
Baker et al. (2024)	Low risk	Low risk	Low risk	Unclear	Low risk	Low risk	Unclear	Low-Moderate
Brown et al. (2005)	Low risk	Low risk	Unclear	low risk	Moderate risk	Low risk	None reported	Low-Moderate
Cottraux et al. (2009)	Low risk	Low risk	Unclear	low risk	High risk	Low risk	None reported	Moderate-High
Davidson et al. (2006)	Low risk	Low risk	Unclear	low risk	Low risk	Low risk	None reported	Low-Moderate
De Jaegere et al. (2019)	Low risk	Low risk	Unclear	Unclear	High risk	Low risk	None reported	Moderate-High
De Jaegere et al. (2024)	Low risk	Unclear	Unclear	Low risk	Low risk	Low risk	Unclear	Moderate
Diefenbach et al. (2024)	Low risk	Low risk	Unclear	Low risk	Moderate risk	Low risk	None reported	Low-Moderate
Fischer et al. (2015)	Low risk	Low risk	Unclear	Unclear	Moderate risk	Low risk	None reported	Moderate
Forkmann et al. (2016)	Low risk	Low risk	Unclear	Unclear	Moderate risk	Low risk	None reported	Moderate
Fu and Li (2016)	Low risk	Low risk	Unclear	Unclear	Low risk	Low risk	Unclear	Moderate
Gandy et al. (2014)	Low risk	Low risk	Unclear	Unclear	Moderate risk	Low risk	None reported	Moderate
Husain et al. (2023)	Low risk	Low risk	Low risk	Low risk	Low risk	Low risk	Unclear	Low
Morley et al. (2014)	Low risk	Low risk	Unclear	Unclear	Moderate risk	Low risk	None reported	Moderate
Mühlmann et al. (2021)	Low risk	Low risk	Unclear	Unclear	Low risk	Low risk	Unclear	Low-Moderate
Nazem et al. (2024)	Unclear	Unclear	Unclear	Unclear	Unclear	Low risk	Unclear	Moderate-High
Palmier-Claus et al. (2025)	Low risk	Low risk	Low risk	Low risk	Low risk	Low risk	Unclear	Low
Rudd et al. (2015)	Low risk	Low risk	Unclear	low risk	Low risk	Low risk	None reported	Low-Moderate
Slee et al. (2008)	Low risk	Low risk	Unclear	Unclear	Moderate risk	Low risk	Unclear	Moderate
Tarrier et al. (2014)	Low risk	Low risk	Unclear	low risk	Moderate risk	Low risk	Unclear	Moderate
Torok et al. (2022)	Low risk	Low risk	Low risk	Low risk	Moderate risk	Low risk	Unclear	Low-Moderate
Tyrer et al. (2003)	Low risk	Low risk	Unclear	Unclear	Moderate risk	Low risk	Unclear	Moderate
van Spijker et al. (2014)	Low risk	Low risk	Unclear	Unclear	Moderate risk	Low risk	Unclear	Moderate
van Spijker et al. (2018)	Low risk	Low risk	Unclear	unclear	Moderate risk	Low risk	Unclear	Moderate
Walton et al. (2020)	Low risk	Low risk	Low risk	Low risk	Low risk	Low risk	Unclear	Low
Weinberg et al. (2006)	Unclear	Unclear	Unclear	Unclear	Moderate risk	Low risk	Unclear	Moderate-High
Weitz et al. (2014)	Unclear	Unclear	Unclear	Unclear	Low risk	Low risk	Unclear	Moderate
Wilks et al. (2018)	Low risk	Low risk	Unclear	Unclear	Moderate risk	Low risk	Unclear	Moderate
Yang et al. (2025)	Low risk	Low risk	low risk	Low risk	Moderate risk	Low risk	None reported	Low-Moderate

Regarding incomplete outcome data, more than half of the studies had moderate or high risk of attrition bias, mainly due to high dropout rates. No selective reporting bias was detected in any of the studies. For “other bias,” most studies did not provide sufficient details, and were thus rated as unclear risk or not reported.

Overall risk assessment indicated that the majority of studies had a moderate or moderately low overall risk of bias; 4 studies (Nazem et al., 2024; Cottraux et al., 2009; De Jaegere et al., 2019; Weinberg et al., 2006) were rated as moderately high risk due to high dropout rates or unclear allocation concealment, and 3 studies (Husain et al., 2023; Palmier-Claus et al., 2025; Walton et al., 2020) were considered to be at low risk of bias. In summary, the overall quality of the included studies was acceptable, with the main limitations being difficulty implementing blinding and the risk of bias from attrition.

### 3.3 Efficacy of CBT for suicidal ideation

**Figure 2:**
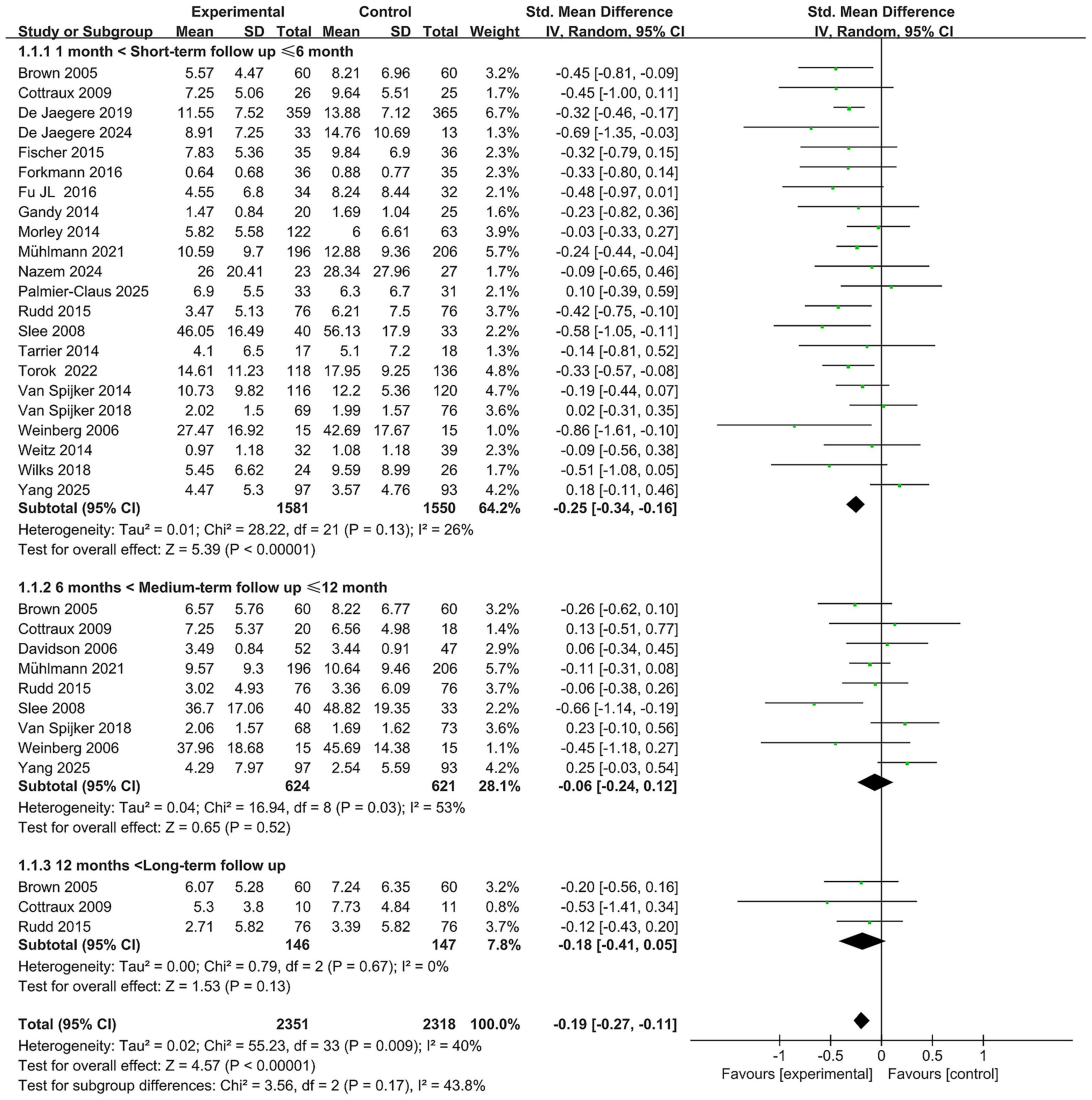
Forest plot for the efficacy of CBT on suicidal ideation.

Twenty-two studies (Brown et al., 2005; Tarrier et al., 2014; Weitz et al., 2014; Nazem et al., 2024; Yang et al., 2025; Cottraux et al., 2009; Rudd et al., 2015; De Jaegere et al., 2019; Torok et al., 2022; De Jaegere et al., 2024; Fischer et al., 2015; Forkmann et al., 2016; Fu and Li, 2016; Gandy et al., 2014; Morley et al., 2014; Mühlmann et al., 2021; Palmier-Claus et al., 2025; Slee et al., 2008; van Spijker et al., 2014; van Spijker et al., 2018; Weinberg et al., 2006; Wilks et al., 2018) (*n* = 3,131) reported the short-term efficacy of CBT on suicidal ideation. Meta-analysis indicated that the CBT intervention group showed a significant reduction in suicidal ideation within 6 months of follow-up (SMD = −0.25, 95% CI: −0.34 to −0.16, *p* < 0.05), with low heterogeneity (*I*^2^ = 26%). The majority of studies showed consistent direction of effect, with post-intervention reductions in suicidal ideation and confidence intervals that largely did not cross zero, supporting the robustness and reliability of the findings. These results indicate that CBT exerts a significant short-term therapeutic effect on suicidal ideation (see Figure 2).

Nine studies (Brown et al., 2005; Yang et al., 2025; Cottraux et al., 2009; Rudd et al., 2015; Davidson et al., 2006; Mühlmann et al., 2021; Slee et al., 2008; van Spijker et al., 2018; Weinberg et al., 2006) (*n* = 1,245) evaluated the medium-term efficacy of CBT. The pooled results indicated a diminished and statistically non-significant effect (SMD = −0.06, 95% CI: −0.24 to 0.12, *p* > 0.05), with moderate heterogeneity (*I*^2^ = 53%, *p* < 0.1). While some studies suggested potential benefits, most confidence intervals crossed zero, indicating inconsistent and unstable outcomes. These findings suggest that CBT does not produce a statistically significant improvement in suicidal ideation at medium-term follow-up. The observed heterogeneity may be attributable to the study by Slee et al. (2008), which recruited individuals with recent self-harming behavior but excluded those with defined psychiatric disorders—a key distinction from other studies that included participants with clearly defined suicidal or self-harming behavior and comorbid psychiatric or personality disorders. After excluding this study (RCT = 8, *n* = 1,172), heterogeneity was not significant (*I*^2^ = 30%, *p* = 0.19), effect size decreased, and the confidence interval narrowed, but the difference remained statistically non-significant (SMD = 0, 95% CI: −0.15 to 0.15, *p* > 0.05) (see Figure 2).

Three studies (Brown et al., 2005; Cottraux et al., 2009; Rudd et al., 2015) (*n* = 293) reported on the long-term efficacy of CBT for suicidal ideation. Results indicated no statistically significant long-term effect (SMD = −0.18, 95% CI: −0.41 to 0.05, *p* > 0.05), with no apparent heterogeneity (*I*^2^ = 0%) (see Figure 2).

### 3.4 Efficacy of CBT for suicide and self-harming behaviors

**Figure 3:**
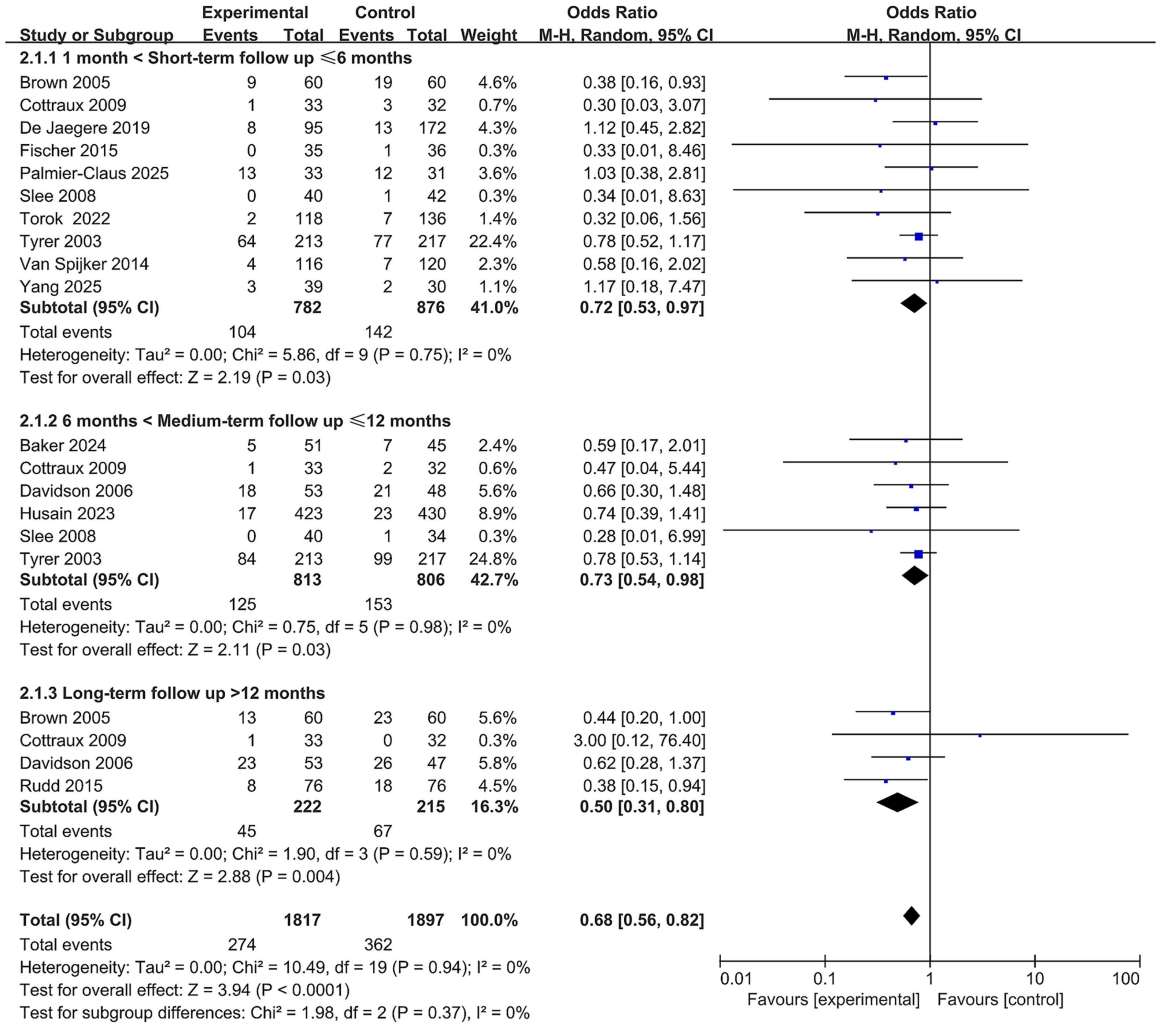
Forest plot for the efficacy of CBT on suicidal and self-harming behaviors.

Ten randomized controlled trials (Brown et al., 2005; Yang et al., 2025; Cottraux et al., 2009; Fischer et al., 2015; Palmier-Claus et al., 2025; Slee et al., 2008; Tyrer et al., 2003; van Spijker et al., 2014), involving a total of 1,658 participants, reported the short-term effects of CBT on suicidal and self-harming behaviors. Meta-analysis revealed a significantly lower incidence of such behaviors in the CBT intervention group compared to the control group (OR = 0.72, 95% CI: 0.53–0.97, *p* < 0.05), with no observed heterogeneity (I^2^ = 0%), indicating that CBT effectively reduces the short-term risk of suicide and self-harm (see Figure 3).

In addition, six studies (Baker et al., 2024; Cottraux et al., 2009; Husain et al., 2023; Davidson et al., 2006; Slee et al., 2008; Tyrer et al., 2003) (*n* = 1,619) and four studies (Brown et al., 2005; Cottraux et al., 2009; Rudd et al., 2015; Davidson et al., 2006) (*n* = 437) assessed the medium- and long-term effects of CBT on suicidal and self-harming behaviors, respectively. Pooled analyses demonstrated that the CBT group had a significantly lower incidence of these behaviors at both follow-up intervals compared to controls, with no substantial heterogeneity detected (see Figure 3).

### 3.5 Efficacy of CBT for depressive symptoms

**Figure 4:**
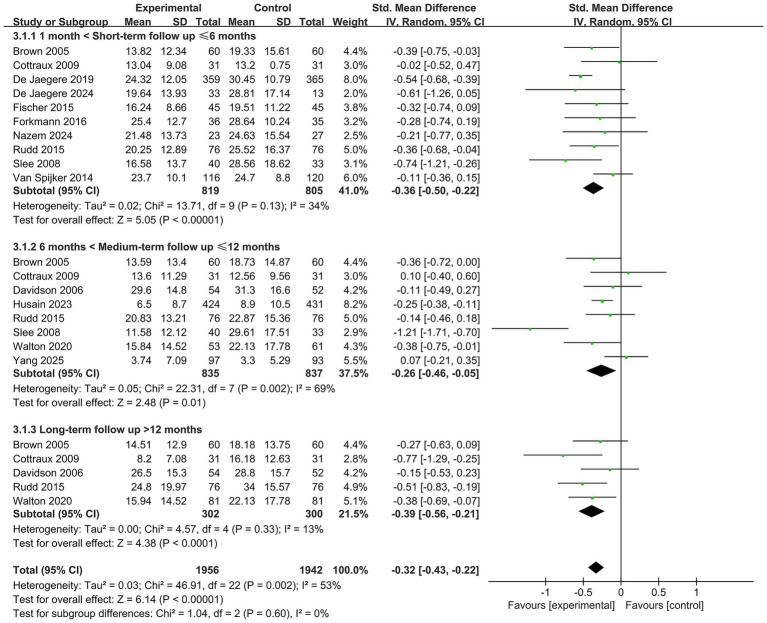
Forest plot for the efficacy of CBT on depressive symptoms.

Ten studies (Brown et al., 2005; Nazem et al., 2024; Cottraux et al., 2009; Rudd et al., 2015; De Jaegere et al., 2019; De Jaegere et al., 2024; Fischer et al., 2015; Forkmann et al., 2016; Slee et al., 2008; van Spijker et al., 2014), encompassing 1,624 participants, investigated the short-term effects of CBT on depressive symptoms. Meta-analysis demonstrated that participants in the CBT group experienced significantly greater reductions in depressive symptoms compared to those in the control group (SMD = −0.36, 95% CI: −0.50 to −0.22, *p* < 0.05), with low heterogeneity (*I*^2^ = 34%), indicating a robust short-term efficacy of CBT on alleviating depressive symptoms (see Figure 4).

Eight studies (Brown et al., 2005; Yang et al., 2025; Cottraux et al., 2009; Rudd et al., 2015; Husain et al., 2023; Davidson et al., 2006; Slee et al., 2008; Walton et al., 2020) (*n* = 1,672) assessed the medium-term effects of CBT. Results showed that CBT remained significantly more effective than control conditions in reducing depressive symptoms (SMD = −0.26, 95% CI: −0.46 to −0.05, *p* < 0.05). However, heterogeneity was relatively high (*I*^2^ = 69%, *p* = 0.002), potentially due to the study by Slee et al. (2008). Upon exclusion of this study (7 RCTs, *n* = 1,599), heterogeneity was substantially reduced (*I*^2^ = 19%, *p* = 0.29), the effect size was attenuated, the confidence interval narrowed, yet the difference remained statistically significant (SMD = −0.18, 95% CI: −0.30 to −0.06, *p* < 0.05) (see Figure 4).

Five studies (Brown et al., 2005; Cottraux et al., 2009; Rudd et al., 2015; Davidson et al., 2006; Walton et al., 2020), involving 602 participants, examined the long-term effects of CBT on depressive symptoms. The meta-analysis indicated that the long-term benefits of CBT remained statistically significant (SMD = −0.39, 95% CI: −0.56 to −0.21, *p* < 0.05), with low heterogeneity (*I*^2^ = 13%) (see Figure 4).

## 4 Discussion

This study employed a systematic review and meta-analytic approach, incorporating 28 randomized controlled trials (RCTs), to comprehensively evaluate the efficacy of cognitive behavioral therapy (CBT) in reducing suicidal ideation, suicidal and self-harming behaviors, and depressive symptoms among adults aged 18–65 years. The findings demonstrated that CBT significantly reduced suicidal ideation in the short term (≤6 months); however, its advantages were not statistically significant at medium- and long-term follow-up. In contrast, CBT was consistently effective in reducing the incidence of suicidal and self-harming behaviors and on alleviating depressive symptoms across short-, medium-, and long-term follow-up periods.

Compared to previous research, the findings of this study differ from those reported by Mewton and Andrews (2016). Mewton et al. observed that only a subset of primary studies supported the effectiveness of CBT for suicidal ideation and behavior, and consequently concluded that only suicide-specific CBT interventions were effective, whereas standard CBT targeting general psychiatric conditions showed no significant impact. Several factors may account for these discrepancies. First, Mewton et al. did not perform subgroup analyses based on follow-up duration. Given that CBT is typically a short-term, structured intervention—often limited to 6 months—aggregating outcomes across varying follow-up periods may have introduced substantial heterogeneity. Second, their review did not systematically assess the quality or heterogeneity of the included studies and included participants over the age of 65, potentially increasing the risk of bias and limiting generalizability.

The present findings also diverge from those of Ghazal et al. (2025), who concluded that the overall effect of CBT on suicidal ideation and behavior was limited, with nearly half of the included RCTs reporting no significant differences between intervention and control groups. Possible explanations for these inconsistent findings include the absence of subgroup analyses based on follow-up duration—despite wide variation in follow-up periods across the 13 included trials—and the exclusive focus on adolescents. Adolescents are influenced by multiple contextual factors, including family, school, and peer dynamics, which may limit the scope and impact of CBT interventions in this population. Therefore, while the present study offers more robust and stratified evidence, further validation through large-scale, high-quality trials is warranted.

### 4.1 Effect of CBT on suicidal ideation

Numerous systematic reviews and meta-analyses have demonstrated that CBT is effective in reducing suicidal ideation. Wu et al., in a synthesis of nine systematic reviews and meta-analyses, reported that CBT was associated with a small-to-moderate reduction in suicidal ideation scores compared to control conditions (SMD = −0.28, 95% CI: −0.36 to −0.21) (Mühlmann et al., 2021). Similarly, Büscher et al., in an individual participant data meta-analysis (IPD-MA) of nine RCTs, confirmed that internet-based CBT (iCBT) significantly reduced suicidal ideation. All effect size indicators favored the iCBT group, with a reliable improvement rate of 40.5% versus 27.3% in the control group, and a lower deterioration rate (2.8% vs. 5.1%) (Büscher et al., 2022). Sander et al. also emphasized the significant effectiveness of iCBT and recommended its prioritization in clinical application (Sander et al., 2023).

However, consistent with the findings of the present study, the therapeutic benefits of CBT appear to be concentrated primarily in the short term following treatment, whereas its medium- and long-term effects remain inconclusive. This pattern—where short-term efficacy surpasses long-term outcomes—has been consistently observed in prior research. For example, Hawton et al. found that among individuals with a history of self-harm, CBT significantly reduced the risk of repeated self-harm within 6 months (OR ≈ 0.54, 95% CI: 0.34–0.85), but this effect was notably attenuated at the 12-month follow-up (OR ≈ 0.80, 95% CI: 0.65–0.98) (Hawton et al., 2016). Likewise, Lu et al., in a study of Chinese adolescents, reported that psychosocial interventions primarily based on CBT had the greatest effect immediately post-treatment, but the benefits began to decline within 1 month (Lu et al., 2023).

There is a growing academic consensus that the superiority of short-term outcomes may be attributed to several factors, including reduced adherence following the conclusion of CBT, changing environmental stressors, and a lack of sustained psychosocial support (Ghazal et al., 2025; Büscher et al., 2022; Wu et al., 2022; Jeong et al., 2023; Sobanski et al., 2021). Some studies have hypothesized that patients who fail to reinforce the therapeutic skills acquired during treatment may gradually lose their coping capacity, resulting in a recurrence of hopelessness and a resurgence of suicidal ideation (Büscher et al., 2022; Wu et al., 2022). Sobanski et al. (2021) further noted that diminishing differences between intervention and control groups during follow-up may also reflect sample heterogeneity and subgroup-specific characteristics, underscoring the need for future research to incorporate longer follow-up durations and structured booster sessions. In summary, the existing evidence supports the short-term efficacy of CBT in mitigating suicidal ideation. To enhance the durability of treatment effects in clinical practice, it is recommended that CBT protocols incorporate follow-up booster sessions, ongoing consolidation of skills, and support from family and social networks to sustain and optimize its suicide prevention impact.

### 4.2 Effect of CBT on suicidal and self-harming behaviors

Cognitive behavioral therapy is used to treat a wide range of mental health concerns. It’s often the preferred type of psychotherapy because it can quickly help client learn about and cope with specific challenges. CBT generally includes fewer sessions than other types of therapy and is done in a structured way. CBT is a useful tool for learning ways to deal with emotional challenges. For example, CBT works as: Manage symptoms of mental health conditions, Keep symptoms of mental health conditions from coming back, treat a mental health condition without medicines and so on.

The results of this study indicate that CBT is effective in reducing the incidence of suicidal and self-harming behaviors across short-, medium-, and long-term follow-up periods. Related studies also support this conclusion. For example, a network meta-analysis identified CBT as the only psychological intervention that significantly reduced the recurrence of suicidal behavior among patients presenting to psychiatric emergency services, compared to standard control conditions (OR ≈ 0.46, 95% CI: 0.25–0.85). Notably, CBT ranked highest among all psychological interventions in terms of effectiveness (*p* ≈ 0.87), with an 87% probability of being the most effective treatment for preventing suicide recurrence (Jeong et al., 2023). Similarly, Hawton et al. also reported that CBT and its derivative approaches significantly reduced the recurrence of self-harm. At six-month follow-up, CBT was associated with a markedly lower risk of repeated self-harm (OR ≈ 0.54, 95% CI: 0.34–0.85), and this effect was sustained at 12 months (OR ≈ 0.80, 95% CI: 0.65–0.98) (Hawton et al., 2016). In a separate systematic review focusing on high-risk incarcerated populations, Pedrola-Pons et al. (2024) further confirmed the efficacy of CBT in reducing both suicidal and self-harming behaviors.

The theoretical foundation underlying CBT’s effectiveness in addressing suicidal behavior lies in its systematic targeting of negative automatic thoughts and maladaptive behavioral patterns. Suicidal behaviors are frequently associated with cognitive distortions (e.g., catastrophizing, hopelessness), dysfunctional coping strategies, and emotional dysregulation. CBT helps individuals identify, challenge, and reframe suicide-related maladaptive cognitions—such as “life is hopeless” or “my problems are unsolvable”—thereby alleviating pervasive feelings of hopelessness and helplessness (Wenzel et al., 2009). In addition, CBT enhances emotional regulation skills, including emotion identification, response delay, and mindfulness techniques, which help individuals tolerate intense negative emotions (e.g., anger, shame, sadness) and reduce the likelihood of impulsive suicidal acts (Linehan et al., 2015).

From a physiological perspective, CBT may also contribute to improved autonomic nervous system regulation and emotional control at the neurobiological level (Mulcahy et al., 2019). Evidence from animal studies and functional magnetic resonance imaging (fMRI) research has demonstrated that CBT restores functional connectivity between the prefrontal cortex and the limbic system, leading to attenuated neural responses to negative stimuli. These findings highlight CBT’s capacity to promote neuroplastic changes in emotion-regulation circuits, thereby reducing vulnerability to suicidal behavior (Wu et al., 2022). In summary, CBT exerts a well-supported and mechanistically grounded therapeutic effect in mitigating suicidal and self-harming behaviors.

### 4.3 Effect of CBT on depressive symptoms

A large number of high-quality meta-analyses and international authoritative guidelines consistently recognize the definite and stable efficacy of CBT in the treatment of depression, making it the gold standard for psychotherapeutic interventions for depressive disorders. Cuijpers et al. and Butler et al. have repeatedly confirmed through large-sample systematic reviews and network meta-analyses that CBT shows moderate to large effect sizes in improving depressive symptoms, increasing remission rates, and restoring functioning in adults, and is also effective among high-risk groups for suicide. Its efficacy is comparable to, or even exceeds, that of pharmacotherapy and other mainstream psychotherapies (Butler et al., 2006; Cuijpers et al., 2020). The latest National Institute for Health Care Excellence guidelines also recommend CBT as the first-line psychological intervention for adults with mild to moderate depression, emphasizing its evidence-based foundation and broad applicability (National Institute for Health and Care Excellence, 2022).

### 4.4 Common and characteristic modules of CBT

Among the 28 included RCTs, cognitive restructuring and behavioral experiments were core modules in suicide intervention and appeared in almost all intervention arms. These routine modules are foundational to CBT; for example, thought records and emotional diaries have become basic components in nearly every CBT study. By encouraging patients to document their negative automatic thoughts, emotional experiences, and behavioral responses, CBT facilitates self-awareness and emotional processing, thereby laying a solid foundation for subsequent cognitive restructuring and behavioral adjustment (Husain et al., 2023; Diefenbach et al., 2024).

Beyond these standard modules, although traditional art therapies—such as painting, music, and crafts—have not yet emerged as the main treatment methods, a variety of expressive, experiential, and mind–body regulation modules with artistic characteristics have been widely integrated into CBT protocols. Role-play and situational simulation have been explicitly applied in some studies, allowing patients to express emotions, improve communication skills, and enhance social competence in a safe environment by simulating real social or crisis scenarios (Brown et al., 2005; Slee et al., 2008; Walton et al., 2020). Some studies incorporated group discussions and emotional expression exercises, encouraging patients to express and process inner emotions through verbal, written, or group interactions, thereby enhancing self-understanding and peer support (De Jaegere et al., 2024; Palmier-Claus et al., 2025). Furthermore, mind–body regulation techniques such as mindfulness meditation, relaxation, and breathing exercises are common in third-wave CBT (such as MBCT, DBT) (De Jaegere et al., 2024; Walton et al., 2020) and iCBT (Nazem et al., 2024; Yang et al., 2025; Mühlmann et al., 2021), helping patients increase present-moment awareness, regulate anxiety and stress, and enhance self-management of emotions. Innovative CBT protocols, such as BMAC (Broad-Minded Affective Coping), use positive psychological imagery, guiding patients to construct positive emotional scenarios through imagination and sharing, further enhancing emotion regulation, and demonstrating a psychological and artistic experiential quality (Palmier-Claus et al., 2025).

It should be noted that although these expressive and experiential modules have not yet developed into stand-alone, art therapy–based interventions, within the multi-module system of CBT, rich experiential exercises—such as writing, role-play, emotional expression, mindfulness, relaxation, and positive imagery—effectively strengthen patients’ self-regulation, interpersonal communication, and crisis coping abilities. These may be among the key mechanisms by which CBT improves suicidal symptoms.

### 4.5 Unexpected findings and their potential causes

It is noteworthy that while CBT demonstrates sustained long-term efficacy in reducing suicidal and self-harming behaviors as well as depressive symptoms, its impact on suicidal ideation appears to be primarily short-term, with no significant effects observed over the long term. This finding seems to contradict the conventional assumption that “improvement in depressive symptoms leads to decreased suicidal ideation, which in turn reduces suicidal and self-harming behaviors.” Several possible explanations may account for this discrepancy:

First, the generation of suicidal ideation may involve more complex mechanisms. For example, Zhang. J et al., in their study of borderline personality disorder patients, found that depression did not have a significant impact on suicidal ideation. Suicidal ideation is often heavily influenced by acute environmental stress, emotional fluctuations, and immediate social support, meaning that sustained improvement in depressive symptoms does not necessarily bring about a sustained reduction in suicidal ideation (Zhang et al., 2017). The focus of CBT intervention varies at different stages of the suicide process. In the early phase of CBT intervention, cognitive restructuring and emotion regulation can quickly reduce intense suicidal thoughts, but such “relief” often relies on frequent support and intensive skills training during the therapy process.

Second, depressive symptoms and recurrent suicidal or self-harming behaviors typically exhibit greater “stability,” which is closely related to individual personality structure and chronic psychological mechanisms (Hawton et al., 2012). The primary focus of CBT is on fundamental cognitive change, problem-solving, and behavioral activation—these core mechanisms aim to adjust such symptoms and behaviors at their roots (Beck et al., 2024). After CBT training, even when new stressors occur, patients’ depressive symptoms and impulsive behaviors have been reconstructed or mitigated, so the recurrence rate can be controlled over a longer term.

Third, the impact of CBT on suicidal ideation often depends on external support and emotional “first aid,” while changes in depression and behavior are more likely to be internalized (Sander et al., 2023). Improvement in depressive symptoms and the reduction of suicidal and self-harming behaviors are largely related to the internalization and generalization of coping skills. After learning to recognize negative thought patterns and behavioral chains, patients can self-regulate over a longer period, even in the absence of intensive support. In contrast, suicidal ideation—especially during a crisis—still requires external support and skills reinforcement, or it is likely to recur.

Fourth, CBT can effectively interrupt the pathway from depressive mood to suicidal and self-harming behaviors through cognitive restructuring and behavioral experiments. However, suicidal ideation, as a form of “emotional polarization response,” often lacks behavioral anchors, and its fluctuation and sensitivity are greater than those of depressive emotions and behavioral symptoms. Therefore, without ongoing intervention and regular introduction of new coping skills, the inhibitory effect of CBT on suicidal ideation is difficult to maintain in the long term.

### 4.6 Limitations and future research directions

Nonetheless, this study has several limitations. First, participant heterogeneity across included studies—spanning individuals with diverse mental and physical health conditions—may have affected the consistency of outcomes. Second, we conducted a systematic assessment of risk of bias across the included studies. Overall study quality was acceptable; the most common concerns were incomplete reporting of randomization and allocation concealment, outcome missingness due to attrition, and reliance on self-report measures for most outcomes, which may introduce measurement bias. These methodological limitations could inflate or destabilize effect-size estimates, particularly in trials with higher attrition. Accordingly, we interpret the main findings with appropriate caution and consider variability in study quality a plausible contributor to between-study heterogeneity. Third, this review focused exclusively on adult populations (aged 18–65) and did not include randomized controlled trials involving children, adolescents, or older adults, thus limiting the generalizability of the findings.

Future research should explore comparative efficacy among different CBT formats—such as traditional face-to-face CBT, iCBT, brief or simplified CBT models, and third-wave approaches (e.g., DBT, ACT, MBCT)—across diverse age groups, cultural contexts, and psychopathological profiles. In addition, identifying key mediators and potential moderators of CBT efficacy will be essential for optimizing intervention content and tailoring strategies to individual needs. Advancing research in these areas will help maximize the clinical and public health impact of CBT in suicide prevention and contribute to the development of comprehensive, evidence-based intervention frameworks. What’s more, The ideation-to-action framework represents that the development of suicidal ideation and the progression from ideation to suicide attempt are different phenomena (Klonsky et al., 2016). This is consistent with the findings of this study, which suggests that CBT has a sustained impact on suicidal behavior and depressive symptoms, but has no effect on suicidal ideation. Future research should distinguish suicide factors from factors that predict suicide attempts and further improve the ideation to action framework.

## 5 Conclusion

Cognitive Behavioral Therapy (CBT) demonstrates significant short-term efficacy (≤6 months) in reducing suicidal ideation among adults. However, its effectiveness in the medium term (6–12 months) and long term (>12 months) does not appear to be significantly greater than that of conventional treatments. Importantly, CBT consistently reduces the incidence of suicidal and self-harming behaviors and alleviates depressive symptoms across short-, medium-, and long-term follow-up periods. These findings have important implications for both clinical practice and public health.

## Data Availability

The original contributions presented in the study are included in the article/supplementary material, further inquiries can be directed to the corresponding author/s.
